# Ultrafine ferroferric oxide nanoparticles embedded into mesoporous carbon nanotubes for lithium ion batteries

**DOI:** 10.1038/srep17553

**Published:** 2015-12-03

**Authors:** Guo Gao, Qiang Zhang, Xin-Bing Cheng, Joseph G. Shapter, Ting Yin, Rongjin Sun, Daxiang Cui

**Affiliations:** 1 Institute of Nano Biomedicine and Engineering, Department of Instrument Science and Technology, Key Laboratory for Thin Film and Microfabrication Technology of Ministry of Education, School of Electronic Information and Electrical Engineering, Shanghai Jiao Tong University, Shanghai, 200240, China; 2 Beijing Key Laboratory of Green Chemical Reaction Engineering and Technology, Department of Chemical Engineering, Tsinghua University, Beijing, 100084, China; 3 School of Chemical and Physical Sciences, Flinders University, Bedford Park, Adelaide 5042, Australia

## Abstract

An effective one-pot hydrothermal method for *in situ* filling of multi-wall carbon nanotubes (CNT, diameter of 20–40 nm, length of 30–100 μm) with ultrafine ferroferric oxide (Fe_3_O_4_) nanoparticles (8–10 nm) has been demonstrated. The synthesized Fe_3_O_4_@CNT exhibited a mesoporous texture with a specific surface area of 109.4 m^2^ g^−1^. The loading of CNT, in terms of the weight ratio of Fe_3_O_4_ nanoparticles, can reach as high as 66.5 wt%. Compared to the conventional method of using a Al_2_O_3_ membrane as template to fill CNT with iron oxides nanoparticles, our strategy is facile, effective, low cost and easy to scale up to large scale production (~1.42 g per one-pot). When evaluated for lithium storage at 1.0 C (1 C = 928 mA g^−1^), the mesoporous Fe_3_O_4_@CNT can retain at 358.9 mAh g^−1^ after 60 cycles. Even when cycled at high rate of 20 C, high capacity of 275.2 mAh g^−1^ could still be achieved. At high rate (10 C) and long life cycling (500 cycles), the cells still exhibit a good capacity of 137.5 mAhg^−1^.

The present annual world energy consumption is about 15 TW (terawatts) and the energy demand is expected to be 30 TW by 2050, and more than 80% of energy demand is met by our traditional non-renewable resources (*e.g.*, oil, coal, and gas)^1^,^2^. Humankind will use up these non-renewable resources faster than physical processes produce them in the near future. Furthermore, the use for electricity production contributes significantly to global warming (e.g., CO_2_ gases)^3^,^4^,^5^. Faced with the real possibility of a global energy crisis, generation of reliable alternative energy devices to satisfy future energy demand is essential to sustain social and economic development. Lead-acid batteries, nickel-metal hydride batteries, solar cells, lithium ion batteries, and supercapacitors are excellent candidates as high energy storage devices to meet the global increasing energy demand^6^,^7^,^8^,^9^,^10^,^11^,^12^,^13^,^14^,^15^,^16^.

In the past 20 years, rechargeable lithium ion batteries (LIBs) have attracted considerable attention due to their many outstanding properties including high energy density, long cycling life, low maintenance, no ‘memory effect’, low self-discharge, wide temperature window and high operating voltage^17^,^18^,^19^,^20^,^21^,^22^. Since the first commercial products manufactured by Sony Corporation, LIBs have been used in applications ranging from portable electronic devices (*e.g.*, laptops, digital cameras and cell phones) and to large-scale devices (*e.g.*, electric cars and bicycles, grass cutter, energy storage devices of solar and wind power, and small medical instruments and equipment)^23^,^24^,^25^. A typical LIB cell consists of an anode (negative electrode, *e.g.*, graphite), a cathode (positive electrode, *e.g.*, LiCoO_2_) and the electrolyte. The anode and cathode are separated by a porous membrane separator and soaked in nonaqueous liquid electrolyte. The charge/discharge mechanism of LIBs is based on the rocking-chair concept. LIBs can offer a large amount of energy as high as 150–200 W h kg^−1^, high power density (~1000 W kg^−1^) and cycling life (~1000 cycles)^17^. The energy density and power density of LIBs are several times higher than Ni-MH, Ni-Cd and Pb acid batteries^26^. In spite of these merits, commercialized LIBs still need much improvement in both energy storage capacities (*e.g.*, energy density and power density) and cycling properties (*e.g.*, capacity retention and Coulombic efficiency) in order to meet the requirements of electrical vehicles and portable electronic devices.

In the recent years, 3D-transition metal oxides (*e.g.*, Fe_2_O_3_, Fe_3_O_4_, SnO_2_, NiO, Co_3_O_4_ and TiO_2_ etc.) with various morphologies have been intensively investigated as potential anode materials for LIBs owing to their special physical/chemical properties^27^,^28^. Wang *et al*. reported a two-step method for the synthesis of NiO nanocone arrays with a three-dimensional network structure^29^. The synthesized NiO nanocone arrays electrodes exhibit a high reversible capacity (1058 mAh g^−1^ after 100 cycles at a rate of 0.4 C) and excellent rate capability (436 mAh g^−1^ at 20 C). TiO_2_ nanostructures have been regarded as one of the ideal anode materials for LIBs because they provide great improvement in safety (lithium insertion/desertion potential is about 1.7 V which can avoid Li electroplating) and near zero volume effect of TiO_2_ lithiation/delithiation process (less than 3% cell expansion which is significantly lower than 200–400% cell expansion of silicon and tin)^30^,^31^. Among the 3D-transition metal oxides, binary iron oxides such as α-Fe_2_O_3_ and Fe_3_O_4_ possess low cost, environmental benignity and high theoretical capacities (1005 mAh g^−1^ for α-Fe_2_O_3_ and 928 mAh g^−1^ for Fe_3_O_4_). These fascinating properties suggest they could be the most promising anode materials for LIBs^32^,^33^,^34^. However, the severe aggregation and huge volume change of iron oxides nanoparticles during the charge/discharge process induce the pulverization of electrodes, and leads to the poor cycling performance and stability^35^. One strategy to enhance the cycling ability was tailoring the iron oxides into hollow structures. Hollow structures can have a thin shell, low density, large internal void and high surface area. The permeable thin shell can effectively shorten the diffusion pathway of Li^+^ and electrons, and increase the electrochemical reaction area. The hollow interior provides extra free space for alleviating the structural strain and tolerating the volume change during the repeated Li^+^ insertion/extraction process. The extra surface area provides the iron oxides more lithium storage sites and a large electrode-electrolyte contacting area for Li^+^ flux across the interface^28^. Koo *et al*. explored the electrochemical performance of hollow iron oxides with a diameter of less than 20 nm^36^. The hollow iron oxides exhibited high capacity (~132 mAh g^−1^ at 2.5 V), 99.7% Coulombic efficiency, superior rate performance (133 mAh g^−1^ at 3000 mAg^−1^) and excellent stability. Due to the low electronic conductivity of iron oxides, most iron oxides particles suffer from rapid capacity fading. To overcome this drawback, the latest strategy to coat a carbon layer on the surface of iron oxides has been developed. It is expected that the carbon shell can act as a cushion barrier to inhibit the aggregation and pulverization of iron oxides, increase the electronic conductivity, and thus increase the cycling performance and stability. Carbon nanotubes (CNT) possess high moduli, high tensile strengths, low densities and good electronic conductivity^37^. Yu *et al*. prepared Fe_2_O_3_-filled CNT using an anodic aluminum oxide (AAO) template method^38^. The Fe_2_O_3_-filled CNT exhibited high reversible capacity, cycling stability (811.4 mAhg^−1^ after 100 cycles) and rate capability, compared with pure Fe_2_O_3_. This method requires a high temperature (heating to 400 °C in Ar atmosphere at 2 °C min^−1^, and held for a further 3.0 h) treatment for precursors, and the removal of AAO template using 5 M NaOH solution. Kopyl *et al*. reported a three-step procedure for the synthesis of CNT filling with iron oxides nanoparticles^39^. This approach includes the preparation of the Al_2_O_3_ membrane with CVD-generated CNT, putting ferrofluid into the CNT and the removal of Al_2_O_3_ membrane by 4.0 M NaOH solution. The filling mechanism is based on capillarity effects using wetting fluids and large diameter CNT (>100 nm) that can be filled with iron oxides nanoparticles. As for this three-step procedure, it involves the use of high cost Al_2_O_3_ membrane, the pore/tubes confinement of Al_2_O_3_ membrane and the removal of Al_2_O_3_ membrane by high concentration NaOH solution. These studies motivated us to explore a new method to fill multi-wall CNT with iron oxides nanoparticles, especially for large scale synthesis.

In this contribution, we report a facile, low cost, and environmental friendly hydrothermal method for the large scale *in situ* filling of multi-wall CNT (diameter of 20–40 nm, length of 30–100 μm) with ultrafine Fe_3_O_4_ nanoparticles (8 ~ 10 nm). The formation process of the mesoporous Fe_3_O_4_@CNT involves two steps, as illustrated in Fig. 1. The advantages of this procedure include neither surfactant such as cetyltrimethyl ammonium bromide (CTAB)/organic solvent, nor templates, such as Al_2_O_3_ membrane; (2) the employed CNT do not need to be cut and opened before filling, which makes it very easy to obtain large scale CNT with uniform morphology and structure, and the prepared CNT filled with Fe_3_O_4_ nanoparticles possess the same length-diameter ratio with that of original CNT; (3) the lack of diameter confinement of the CNT (e.g., the method of using Al_2_O_3_ membrane often needs larger diameter of CNT (>50 nm)^39^,^40^. means the CNT can be filled with more Fe_3_O_4_ nanoparticles regardless of the diameter of CNT; (4) the loading level of CNT, in terms of the weight ratio of Fe_3_O_4_ nanoparticles filled in CNT, can reach 66.5 wt% which is significantly higher than the highest reported value (51.8 wt%)^38^. This large amount of ultrafine Fe_3_O_4_ nanoparticles filled into the CNT backbone enhances the electrochemical reactivity and mechanical integrity of the electrode during the repeated charge/discharge process; (5) the prepared Fe_3_O_4_@CNT exhibited mesoporous properties. In theory, such hybrid structures are expected to greatly improve the electrochemical performance because of their unique structures, relatively high specific surface area and porosity. The highly flexible and conductive CNT backbone provide a three-dimensional network to facilitate the electron transfer, and to provide a large contact area for higher Li^+^ diffusion between the electrode and electrolyte^41^. When evaluated for lithium storage capacity, the capacity of the prepared Fe_3_O_4_@CNT remained at 358.9 mAh g^−1^ after 60 cycles at a rate of 1.0 C. Even when cycled at high current rate of 20 C, acceptable capacity of 275.2 mAhg^−1^ was achieved. At high rate (10 C) and long life cycling (500 cycles), the cells still exhibited a similar capacity of 137.5 mAh g^−1^, indicating the introduction of mesoporous carbon shell (multi-wall CNT) can greatly enhanced the electrochemical performance of Li storage.

## Experimental Section

### Synthesis of multi-wall CNT

The muti-wall CNT were mass produced by our previous procedure using a nano-agglomerate fluidized bed reactor method^42^. This procedure involves the design of catalyst, agglomeration control, the fluidization hydrodynamic process, and the large scale fabrication of CNT in an industrial reactor, and routine purification.

### Oxidation of muti-wall CNT

In a typical procedure, 1.0 g multi-wall CNT were added in a mixed acid solution containing 10.0 mL HNO_3_ and 30.0 mL H_2_SO_4_ at room temperature. The mixture was stirred for 10.0 min, and then the mixture was heated to 80 °C for 20.0 min. After that, the mixture was naturally cooled to room temperature. Then, the products were filtered, washed by deionized water three times and finally dried in a freeze-drying apparatus for 24.0 h.

### Synthesis of mesoporous Fe_3_O_4_@CNT

Typically, 0.9 g oxidized CNT was mixed with ferric citrate and FeSO_4_·7H_2_O solution in the mole ratio of 1:2. When the mixture was stirred for 5.0 min, 0.10 g vitamin C (Vc) was added. The effect of Vc was to inhibit the oxidation of Fe^2+^ during the reaction process. The suspension was further mixed for 20 min. Then, the pH of the suspension was adjusted to 10.0 using a by NaOH solution (0.4 M). Then the mixed solution was transferred into a Teflon-lined stainless autoclave and heated to 180 °C for 20.0 h. After reaction, the obtained mesoporous Fe_3_O_4_@CNT was separated by a magnet, washed by deionized water five times and dried in a freeze-drying apparatus for 24.0 h.

### Characterization

The composition of the synthesized mesoporous Fe_3_O_4_@CNT was determined with an X-ray powder diffractometer (XRD, Rigaku, Japan) using Cu K_α_ radiation at 1.5418 Å at a scanning rate of 5° min^−1^. Scanning electron microscopy (SEM, FEI-Sirion 200), transmission electron microscopy (TEM, JEM-2010), high-resolution transmission microscopy (HRTEM) and selected area electron diffraction (SAED) were used to observe the crystal structure and sizes. Atomic force microscopy (AFM, E-Sweep) was used to obtain three-dimensional images of the as-prepared mesoporous Fe_3_O_4_@CNT. The thermal analysis was determined by a thermogravimetric analyzer (Pyris 1 TGA, PerkinElmer, USA) under N_2_ atmosphere and in air, respectively, at a heating rate of 10 °C min^−1^ from 20 °C to 900 °C. The specific surface area and pore distribution were analyzed by Brunauer-Emmett-Teller (BET) tests using ASAP 2020 (Micromeritics Instuments) analyzers. The magnetic property of the synthesized Fe_3_O_4_@CNT was evaluated by a vibrating sample magnetometer (VSM, Lakeshore 736, USA). The Fourier transform infrared spectroscopy (FTIR) was done by a Perkin Elmer Paragon-1000 spectrometer.

### Electrochemical evaluation of mesoporous Fe_3_O_4_@CNT for LIBs

The electrochemical experiments using the synthesized mesoporous Fe_3_O_4_@CNT were performed using CR2025 coin cells. The working electrode was prepared by mixing the active materials, acetylene black and polyvinylidene fluoride (PVDF) with weight ratios of 80:10:10 in N-methyl-2-pyrrolidone solvent. Coin cells were assembled in an argon-filled glovebox in the presence of an oxygen scavenger and sodium drying agent. The loading amount of active material is 0.82 mg cm^−2^. The mesoporous Fe_3_O_4_@CNT composites act as the working electrode, metallic lithium as counter/reference electrode, 1.0 M LiPF_6_ in ethylene carbonate, diethyl carbonate and ethylmethyl carbonate (EC/DMC/EMC, volume ratio of 1:1:1) electrolyte, and Celgard 2400 polypropylene as separator. The capacitance is calculated based on Fe_3_O_4_.

## Results and Discussion

**Figure 2:**
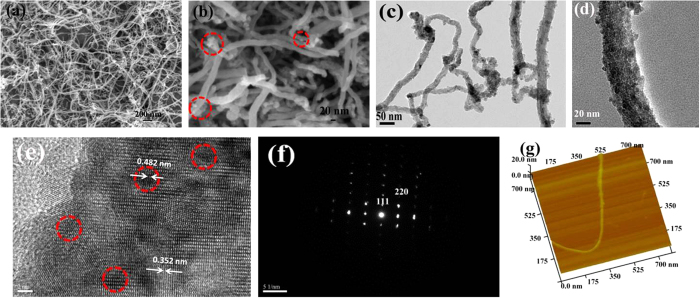
SEM images (a,b), TEM images (c,d), HRTEM image (e), SAED pattern (f), and AFM image (g) for the synthesized mesoporous Fe_3_O_4_@CNT.

The crystallographic structure and phase composition of the synthesized Fe_3_O_4_@CNT are determined by X-ray powder diffraction (XRD), as shown in Figure S1. All of the diffraction peaks can be assigned to the inverse cubic spinel structure Fe_3_O_4_ (JCPDS No. 19-629) and carbon nanotubes. Two peaks at 2θ = 26.5° and 40.6° can be attributed to the (002) and (110) planes of CNT, respectively. The diffraction peak at 40.6° confirms the multi-wall structure of carbon nanotubes. The broad peaks of Fe_3_O_4_ indicated that the size of Fe_3_O_4_ particles is very small. According to the (311) plane data and applying the Scherrer’s diffraction equation (D = Kλ/βcosθ), the average crystalline size is approximately 10.4 nm. The rather sharp diffraction peaks reveal that the Fe_3_O_4_ nanoparticles have a relatively high crystallinity. The Brunauer-Emmett-Teller (BET) surface areas and porous structure of Fe_3_O_4_@CNT were investigated by nitrogen adsorption/desorption isotherms, as shown in Figure S2. The synthesized Fe_3_O_4_@CNT has type IV nitrogen adsorption and desorption isotherm^43^, suggesting the existence of mesoporous which may contribute to a relatively high BET surface area. Our results indicate that the BET surface area of the Fe_3_O_4_@CNT was 109.4 m^2^g^−1^, which is much higher than that of the commercial Fe_3_O_4_ (~2.0 m^2^g^−1^). Although large surface area is detrimental to Coulombic efficiency^44^, based on previous work, the surface area of our synthesized Fe_3_O_4_@CNT was in a reasonable range. The SnO_2_/CNT composites with a large surface area (180.3 m^2^g^−1^) have been demonstrated to be effective as anodes for lithium ion batteries^45^. The CNT/V_2_O_5_ composites with a surface area (80 m^2^g^−1^) also exhibited high-performance for lithium ion electrodes^46^. The pore size distribution obtained through the Barrett-Joyner-Halenda (BJH) method indicates that most pores have the sizes of ~2.5 nm, which is attributed to the surface carbon layers of Fe_3_O_4_@CNT. Figure 2a shows a panoramic image of mesoporous Fe_3_O_4_@CNT by the SEM observation. It is evident that the samples contain numerous dispersed one-dimensional Fe_3_O_4_@CNT, and the length of mesoporous Fe_3_O_4_@CNT is 30–100 μm. Figure 2b shows that the diameter of mesoporous Fe_3_O_4_@CNT varies from 20 to 40 nm. We also observed that a small number of Fe_3_O_4_ nanoparticles decorated with the outer surface of CNT. The formation of mesoporous Fe_3_O_4_@CNT structure was further confirmed by TEM. Figure 2c illustrates the backbones of CNT are uniformly filled with Fe_3_O_4_ nanoparticles, and the *in situ* filling process does not influence the morphology of CNT significantly. This means that the size of the filling Fe_3_O_4_ nanoparticles was very small. A higher magnification in Fig. 2d shows that the Fe_3_O_4_ nanoparticles have a diameter of ~8 nm. The inner space of CNT is completely filled with such ultra-small Fe_3_O_4_ nanoparticles. Consistent with our SEM image, the outer surface of CNT is decorated with some small-sized Fe_3_O_4_ nanoparticles. The outline of the structure of the Fe_3_O_4_@CNT could be clearly observed by the HRTEM, as shown in Fig. 2e. The dark areas (e.g., red circle) are the Fe_3_O_4_ components, and the grey areas are the carbon shell of CNT. The Fe_3_O_4_ nanoparticles have a lattice-fringe spacing of 0.482 nm, which corresponds to the (111) plane of cubic Fe_3_O_4_. The fringe spacing of the carbon shell of CNT is 0.352 nm. It clearly observed that the cavities of CNT are filled by Fe_3_O_4_ nanoparticles. These defects on the surface of CNT are attributed to the oxidation of CNT in the mixed acid solution, and the removal of oxygen-containing groups during the hydrothermal process. As for the dark areas, the SAED rings (Fig. 2f) confirm the monocrystalline nature of Fe_3_O_4_ nanoparticles. The AFM image in Fig. 2g shows the Fe_3_O_4_@CNT has a well-defined edge and structural integrity. The height of Fe_3_O_4_@CNT is about 20 nm. Some small-sized Fe_3_O_4_ nanoparticles are seen on the outside surface of CNT.

**Figure 3:**
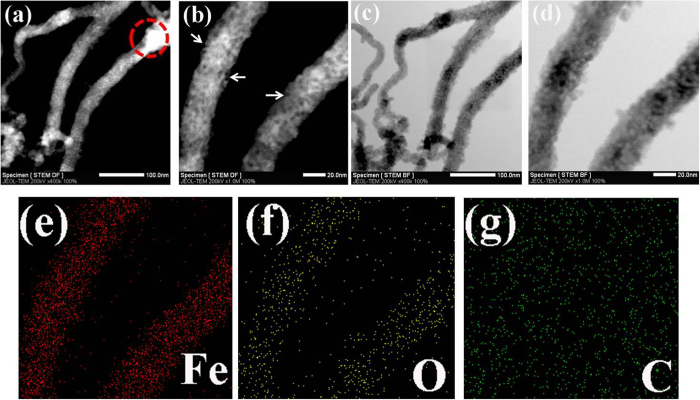
STEM dark field images (a,b), bright field images (c,d), the EDS elemental mapping analysis of Fe (e), O (f) and C (g) for the synthesized mesoporous Fe_3_O_4_@CNT.

The uniform distribution of Fe_3_O_4_ nanoparticles in the CNT was also confirmed by scanning transmission electron microscopy (STEM) observations and energy dispersive spectroscopy (EDS) elemental mapping analysis. The STEM dark field image in Fig. 3a shows the Fe_3_O_4_@CNT appear bright on the dark background. The brightness of Fe_3_O_4_@CNT is relatively uniform, indicating the CNT are uniformly filled with Fe_3_O_4_ nanoparticles. At the edge of CNT (*e.g.*, red circle), the brightness is higher than other areas. The reason is that the edge of CNT has much more oxygen-containing groups which facilitate the aggregation of Fe_3_O_4_ nanoparticles. A higher magnification dark field image of Fig. 3b clearly shows the Fe_3_O_4_ nanoparticles are uniformly filled in the CNT. Some defects (white arrow) on the surface of CNT are also observed. The STEM bright field image of Fig. 3c shows the Fe_3_O_4_@CNT appear dark on the bright background. The uniform darkness of Fe_3_O_4_@CNT confirms the loading level of Fe_3_O_4_ nanoparticles in CNT is complete and high. Figure 3d shows a few Fe_3_O_4_ nanoparticles are attached on the outside surface of CNT backbone. STEM dark and bright images confirm that our hydrothermal method is suitable for the large scale filling of CNT with ultrafine Fe_3_O_4_ nanoparticles regardless of the diameter confinement effect of CNT. Furthermore, the filling process maintains the mechanical integrity of CNT backbone. The uniform distribution of Fe_3_O_4_ in the CNT was further confirmed by the EDS elemental mapping. It is evident that the Fe element (Fig. 3e) and O element (Fig. 3f) were evidently observed. The C element (Fig. 3g) arising from Fe_3_O_4_@CNT was not evident because the samples were dispersed on the carbon grid, and the carbon substrate will weaken the relative contrast intensity of carbon element, which also leads to the EDS mapping area larger than that in the TEM image. From the EDS mapping results, the Fe_3_O_4_ nanoparticles (Fe and O elements) were uniformly distributed in the CNT without strong agglomeration. Such uniform distribution of Fe_3_O_4_ nanoparticles in the CNT was beneficial for improving the electrical conductivity, enhancing the cyclic stability and rate capability, and tolerating the volume expansion of Fe_3_O_4_ nanoparticles during the lithium ion insertion/extraction process. The mesoporous structure of the Fe_3_O_4_@CNT facilitates the electrolyte penetratinge the carbon shell of CNT, promoting rapid lithium ion diffusion and resulting in the high accessibility of the active materials.

**Figure 4:**
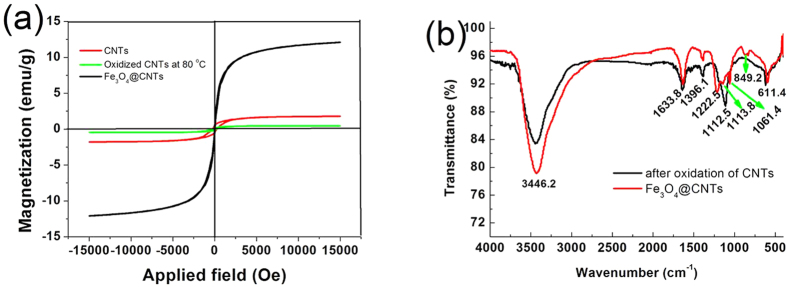
Magnetization loops of CNT, oxidized CNT by mixed acid solution at 80 °C and the synthesized mesoporous Fe_3_O_4_@CNT (a) and FTIR spectra for the oxidized CNT and mesoporous Fe_3_O_4_@CNT (b).

The magnetic properties of the synthesized Fe_3_O_4_@CNT reflect the filling level of Fe_3_O_4_ nanoparticles in the CNT, which is related to electrochemical performance of lithium ion battery. Herein, the magnetic performance of the CNT, the oxidized CNT, and the Fe_3_O_4_@CNT was evaluated by a VSM analyzer at room temperature. Figure 4a presents the magnetic hysteresis curves for the CNT, oxidized CNT and the synthesized Fe_3_O_4_@CNT composites. As for paramagnetic materials, the magnetic moment of the entire crystallite tends to align with the magnetic field. As for superparamagnetic materials, the values of remanent magnetization and coercivity are all zero, and the two magnetic hysteresis curves overlap and go through the zero point, suggesting the synthesized CNT are paramagnetic materials. The saturation magnetization and remanent magnetization are 1.78 and 0.45 emu g^−1^, respectively. The coercivity is found to be 251.7 Oe. The magnetic behavior of CNT arises from the use of metal catalysts during the catalytic growth. After the CNT were oxidized by the mixed acid solution, the saturation magnetization (0.48 emu g^−1^), remanent magnetization (0.08 emu g^−1^) and coercivity (197.9 Oe) all decreased. This was attributed to the partial dissolution of residual metal catalyst in the CNT. When the CNT were filled with ultra-small Fe_3_O_4_ nanoparticles, the remanence and coercivity are negligible. This evolution shows that the synthesized mesoporous Fe_3_O_4_@CNT exhibit a superparamagnetic behaviour due to the presence of the ultra-small Fe_3_O_4_ nanoparticles. The saturation magnetization is found to be 12.1 emu g^−1^. After oxidation of CNT by the mixed acid solution, the surface of CNT contains many oxygen-containing active groups (e.g., −COO^−^, −OH and −C=O). These active groups may serve as the nucleation sites for the Fe^2+^ in the reaction solution. The presence of Fe^2+^ also may lead to the reduction of the oxygen-containing groups during the hydrothermal process which can be confirmed by FTIR spectroscopy, as shown in Fig. 4b. The FTIR spectra were carried out in the wavenumber range of 400–4000 cm^−1^. The intense peak at 3446.2 cm^−1^ was attributed to the O-H stretching vibration. The peaks at 1633.8 cm^−1^ was assigned to the C=O stretching vibrations from carbonyl and carboxylic groups. The peak at 1396.1 cm^−1^ can be assigned to the skeletal vibrations of CNT^47^. The peak at 1112.5 cm^−1^ can be attributed to the C−O−C stretching vibrations^48^, and the peak at 611.4 cm^−1^ may be due to the residual metal catalyst in the CNT. When the oxidized CNT were filled with Fe_3_O_4_ nanoparticles, the peak of C−O−C group becomes a very weak peak (1113.8 cm^−1^), and has a slight shift. The characteristic absorption peak of oxygen-containing C−O−C group decreased dramatically indicates that the oxidized CNT have been reduced. The peak at 849.2 cm^−1^ can be attributed to the vibrations of Fe-O^49^.

**Figure 5:**
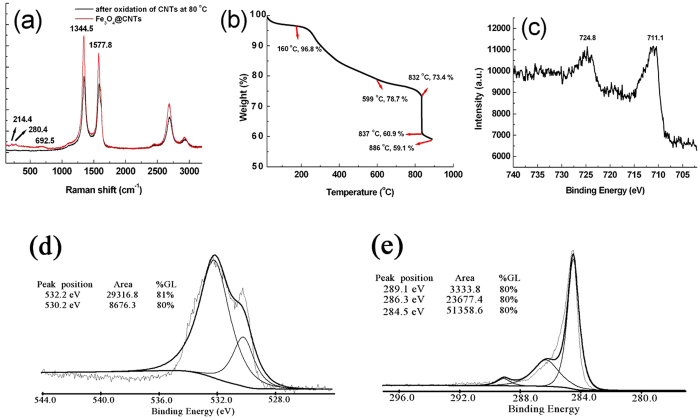
Raman spectra (a), TGA measurement under N_2_ protection (b), XPS spectra of Fe2p region (c), O1s region (d) and C1s region (e) for the synthesized mesoporous Fe_3_O_4_@CNT.

The Raman spectra of the oxidized CNT and the synthesized mesoporous Fe_3_O_4_@CNT are shown in Fig. 5a. It is obvious that there are two strong peaks at 1344.5 cm^−1^ and 1577.8 cm^−1^, respectively. The ‘D’ band at 1344.5 cm^−1^ is related to the defects and disorders in the hexagonal framework of the nanotuble walls^50^. So far, the contribution to the ‘D’ band from the defects of nanotube walls and other forms of carbon (*e.g.*, rings) is still not completely understood. The ‘G’ band appeared at 1577.8 cm^−1^, which is related to the vibration of sp^2^-bonded carbon atoms in a two-dimensional hexagonal lattice. This peak corresponds to the E_2g_ graphite-like tangential mode. As for the synthesized mesoporous Fe_3_O_4_@CNT, three peaks characteristic of iron oxides appeared at 214.4, 280.4, and 692.5 cm^−1^, which were assigned to the E_g_, T_2g_ and A_1g_ vibration modes, respectively^51^,^52^. The intensity ratio of the ‘D’ to ‘G’ band is about 1.13 and 1.18 for the oxidized CNT and the mesoporous Fe_3_O_4_@CNT, respectively. Generally, the intensity ratio of ‘D’ peak (*I*_*D*_) to ‘G’ peak (*I*_*G*_) can be used to estimate the defect degree of multi-wall CNT. The increase of *I*_*D*_/*I*_*G*_ ratio indicates that after filling the oxidized CNT with Fe_3_O_4_ nanoparticles in the hydrothermal system, the surface defects of CNT have a slightly increase. On the other hand, when the oxidized CNT were filled with Fe_3_O_4_ nanoparticles, all the intensities of ‘D’ peak and ‘G’ peak have a further increased compared with the oxidized CNT, indicating the defect density on theof carbon materials has decreased. In order to evaluate the surface properties and loading level of CNT by the Fe_3_O_4_ nanoparticles, the synthesized mesoporous Fe_3_O_4_@CNT were measured by TGA under N_2_ atmosphere (Fig. 5b) and in air (Figure S3), respectively. From Fig. 5b, we can see that there was a tiny weight loss (3.2%) in the temperature range of 20–160 °C. This weight loss can be assigned to the loss of adsorbed water on the surface of mesoporous Fe_3_O_4_@CNT. An approximately 18.1% weight loss occurring in the temperature range from 160–599 °C was attributed to the decomposition of labile oxygen functional groups from CNT layer. In the temperature interval of 599–832 °C, the weight loss (5.3%) was ascribed to the decomposition of stable oxygen functional groups from the CNT layer. When the temperature is over 832 °C, the TGA curve has a sharp decrease. The significant weight loss (12.5%) takes place between 832 °C and 837 °C can be attributed to the removal of very-stable oxygen functional groups from CNT layer. With the further increase of temperature (837–886 °C), a slow mass loss can be observed. The tiny weight loss (1.8%) may be attributed to the much more stable oxygen functional groups from CNT. From Figure S3, we can calculate the loading level of CNT, in terms of the weight ratio of Fe_3_O_4_ nanoparticles filled/embedded in CNT and it was found that the loading level can be as high as ~66.5 wt%. Figure 5c shows the Fe2p high-resolution X-ray photoelectron spectra (XPS) of the synthesized mesoporous Fe_3_O_4_@CNT. There is no evident satellite peak at 719.2 eV, which is the characteristic peak for Fe_2_O_3_, indicating the CNT are filled by largely with Fe_3_O_4_ nanoparticles. Two broad peaks of Fe2p_3/2_ and Fe2p_1/2_ are observed at 711.1 and 724.8 eV, respectively, indicating the formation of iron oxide of Fe (II) and Fe (III), *i.e.*, Fe_3_O_4_. The O1s spectrum is shown in Fig. 5d. There are two peaks in the XPS spectrum. The sharp peak at 530.2 eV originated from the oxygen in Fe_3_O_4_, and the shoulder at 532.2 eV was assigned to the oxygen in CNT^53^. The carbon element in the Fe_3_O_4_@CNT was confirmed by the C1s spectrum, as shown in Fig. 5e. The peak at 284.5 eV is attributed to the C-C bond. The peaks at 286.3 eV and 289.1 eV are the characteristic peaks for C-O and C=O bonds, respectively^54^. The XPS results show the surface of mesoporous Fe_3_O_4_@CNT still possess many oxygen-containing groups.

**Figure 6:**
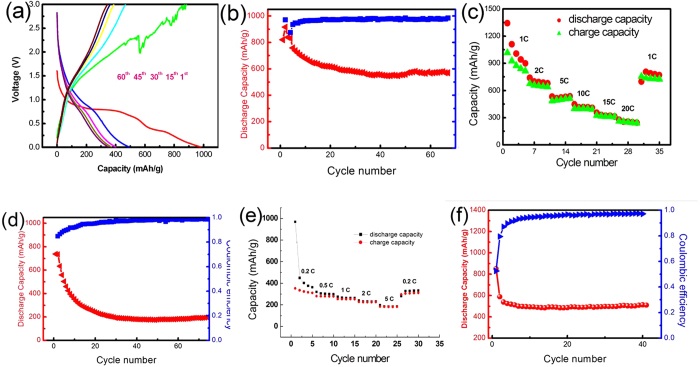
Charge-discharge profiles of the synthesized mesoporous Fe_3_O_4_@CNT (a), cycling performance and Coulombic efficiency of the cell at 0.5 C rate (b) and the rate performance of mesoporous Fe_3_O_4_@CNT at different current densities (c), cycling performance of CNT electrode at 0.5 C (d), rate performance of CNT electrode at different current densities (e) and cycling performance of pure Fe_3_O_4_ nanoparticles electrode at 0.5 C (f).

The electrochemical performance of the synthesized mesoporous Fe_3_O_4_@CNT was evaluated as anodes for LIBs in a CR2025 coin-type cells. Figure 6a shows representative discharge/charge voltage profiles of the synthesized mesoporous Fe_3_O_4_@CNT at a current density of 1.0 C within the cut-off voltage window of 0.01–3.0 V. As for the first discharge profile, there is a voltage plateau at 0.8 V and then the voltage plateau followed by a long slope. This voltage plateau reveals the reduction of Fe^3+^ to Fe^0^, and the formation of amorphous Li_2_O as well as the irreversible reaction with the electrolyte. The sloping tail (referred to the additional plateau at ~0.5 V) below the conversion regime can be assigned to the formation of gel-like film and/or interfacial lithium storage^55^,^56^. The initial discharge and charge capacities are found to be 981.4 and 881.9 mAhg^−1^, respectively. The irreversible capacity loss of about 10.2% may be ascribed to the conversion reaction of the Li-Fe-O compound to Fe and Li_2_O, and the formation of inorganic solid electrolyte interface (SEI) film, electrolyte decomposition and the reaction of lithium ions with oxygen-containing groups in the CNT layer^57^. At the following cycles, the discharge capacity of mesoporous Fe_3_O_4_@CNT electrode in the 15^th^, 30^th^, 45^th^ and 60^th^ cycle is found to be 490.8, 397.8, 376.3 and 358.9 mAh g^−1^, respectively. In the charge process, the charge capacity is found to be 471.3, 389.3, 363.3 and 343.8 mAh g^−1^ in the 15^th^, 30^th^, 45^th^ and 60^th^ cycle, respectively. After 60 cycles, the discharge and charge profiles nearly overlap, suggesting that the mesoporous Fe_3_O_4_@CNT electrode has a good electrochemical cycling ability. The cyclic stability of the mesoporous Fe_3_O_4_@CNT was evaluated at a rate of 0.5 C, as shown in Fig. 6b. From the 15^th^ cycle onwards, the discharge capacities of the mesoporous Fe_3_O_4_@CNT gradually decreased from 622.7 mAhg^−1^ to 571.1 mAhg^−1^ within 70 cycles with a high Coulombic efficiency of about 95–97%. For pure Fe_3_O_4_ nanoparticles, the specific capacity decreased significantly and retained only about 200 mAhg^−1^ at a current density of 200 mAg^−1^ after 50 cycles^58^. The results indicate that the synthesized mesoporous Fe_3_O_4_@CNT exhibit good structural stability. Figure 6c shows the rate capability of the mesoporous Fe_3_O_4_@CNT at different current densities. It is obvious that the mesoporous Fe_3_O_4_@CNT delivered very high capacities of 684.1, 541.5, 432.2 and 358.9 mAh g^−1^ at the current densities of 2.0, 5.0, 10 and 15 C, respectively. Even as a high current density of 20 C, the capacity still remains as high as 275.2 mAh g^−1^, suggesting the introduction of mesoporous CNT may significantly enhance the rate capability. When returning to the initial current density of 1.0 C, the mesoporous CNT electrode returns to a relatively high capacity (826.4 mAh g^−1^) compared with the original capacity (939.6 mAhg^−1^). This confirms that the mesoporous CNT electrode can keep its integrity during the repeated cycling process. In order to support the superior electrochemical performance of the mesoporous Fe_3_O_4_@CNT composites, the electrochemical data of CNT electrode and pure Fe_3_O_4_ nanoparticles electrode are provided to compare with that of the Fe_3_O_4_@CNT composites. Figure 6d shows the cycling performance of CNT electrodes at 0.5 C. It can be seen that there is a large capacity loss between the first and second cycles for the pure CNT electrodes because of the formation of SEI layer on the surface of electrode. After 30 cycles, the capacity is only ~188.1 mAh g^−1^ and maintains this value until 75 cycles. We also investigated the rate performance of CNT electrodes under different current densities, as shown in Fig. 6e. It can be seen that the CNT electrodes delivered low capacities of 410.3, 305.4, 254.1, 226.1 and 186.7 mAh g^−1^ at the current densities of 0.2, 0.5, 1.0, 2.0 and 5.0 C, respectively. As for mesoporous Fe_3_O_4_@CNT electrodes, the capacity can reach as high as 684.1 and 541.5 mAhg^−1^ at the current density of 2.0 and 5.0 C which were about 3 times that of CNT electrode. When the CNT electrode returns to the initial current density of 0.2 C, the capacity can only return to 328.9 mAhg^−1^. Figure 6f shows the cycling performance of pure Fe_3_O_4_ nanoparticles electrode at 0.5 C. The synthetic procedure of Fe_3_O_4_ nanoparticles used for LIBs test was similar to the synthesis of Fe_3_O_4_@CNT, only the oxidized CNT were not added. The morphology of Fe_3_O_4_ nanoparticles was shown in Figure S4. From Fig. 6f, it can be seen that the capacity has a quickly decreased between the first and the third cycles for the pure Fe_3_O_4_ nanoparticles electrode. The initial discharge capacity was 862.6 mAhg^−1^ which is slightly lower than that of the Fe_3_O_4_ theoretical capacity value (928 mAh g^−1^). The capacity dramatically decreased to 539.8 mAhg^−1^ after 3 cycles, and maintaining about 485.4 mAh g^−1^ after the following cycling. The electrochemical data of CNT electrode and pure Fe_3_O_4_ nanoparticles electrode indicate that the synthesized mesoporous Fe_3_O_4_@CNT composites exhibited good electrochemical performance.

**Figure 7:**
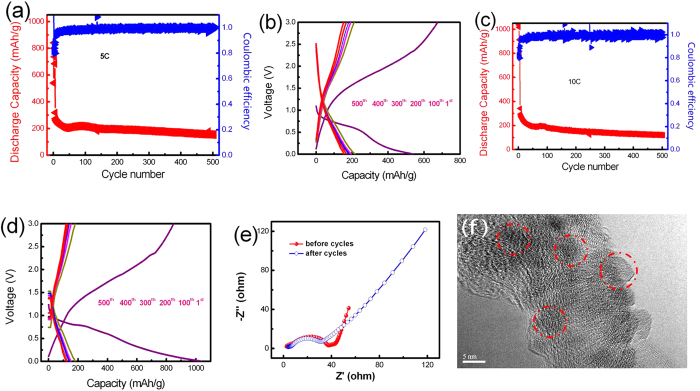
Cycling performance of the synthesized mesoporous Fe_3_O_4_@CNT at the rate of 5 C (a) and 10 C (c), and charge-discharge profiles of the cell at the rate of 5 C (b) and 10 C (d), Nyquist plots of ac impedance spectra of the mesoporous Fe_3_O_4_@CNT before and after 50 cycles at a rate of 2.0 C in the frequency range between 100 kHz and 10 mHz (e), and HRTEM image of the mesoporous Fe_3_O_4_@CNT after 50 cycles at a rate of 2.0 C (f).

In order to investigate the rate performance of the synthesized mesoporous Fe_3_O_4_@CNT, cells were cycled at high current densities (5.0 and 10 C) over 500 cycles. Figure 7a shows the long-term cycling performance under high current density of 5.0 C. The capacity decreased in the first 10 cycles, which was attributed to the formation of SEI film. After 30 cycles, the capacity stabilized at around 231.1 mAhg^−1^. The capacity decayed gradually to 188.3 mAhg^−1^ after 500 cycles. Figure 7b shows the charge-discharge profiles of the mesoporous Fe_3_O_4_@CNT for the 1^st^, 100^th^, 200^th^, 300^th^, 400^th^ and 500^th^ cycle at a rate of 5.0 C. It is clearly observed that the capacity curves almost overlap after 100 cycles, and the capacity maintain around 180 mAh g^−1^ at the following cycles. The relatively low capacity of the mesoporous Fe_3_O_4_@CNT electrode may originate from the CNT components, not the ultrafine Fe_3_O_4_ nanoparticles. With the further increase of current density, the mesoporous Fe_3_O_4_@CNT still exhibit a good electrochemical performance, as shown in Fig. 7c. At 10 C, while the capacity decreased in the first 10 cycles, while the capacity stabilized at around 215.2 mAh g^−1^ after 30 cycles, and remained at around 137.5 mAh g^−1^ after 500 cycles. Figure 7d shows the charge-discharge profiles of this composite at the 1^st^, 100^th^, 200^th^, 300^th^, 400^th^ and 500^th^ cycle under a rate of 10 C. The capacity was found to be 182.6 mAh g^−1^ at the 100^th^ cycle, and stabilized at around 140 mAhg^−1^ after the following cycles. During the test of charge-discharge, one cell was used for 5.0 C and another cell is used for 10.0 C. The individual difference of cells may result in the difference of charge-discharge profiles, especially for the first cycle profile.

Due to the unique mesoporous structure of the CNT and the presence of large amount monodisperse Fe_3_O_4_ nanoparticles filled in CNT, our materials exhibit good cycling capability, stability and rate performance. It is widely accepted that the specific capacity and cycling performance of iron oxides (*e.g.*, α-Fe_2_O_3_ and Fe_3_O_4_) are closely related with their shapes and microstructures. Our previous study shows that α-Fe_2_O_3_ microdisks exhibited good cyclic stability and rate performance for LIBs^59^. The result demonstrated that the disk-like structure was facilitating the transfer of Li^+^ ions and electrons. Among the promising metal oxides for LIBs anode materials, magnetite (Fe_3_O_4_) has high theoretical capacity, which is about three times than that of conventional graphite. However, the specific capacities of pure Fe_3_O_4_ decay readily because: (1) the chemical reaction of Fe_3_O_4_ in lithium storage (Fe_3_O_4 _+ 8 Li^+^ + 8e^−^ ↔ 3Fe + 4Li_2_O) induces structure pulverization after the lithium inclusion; (2) the generated Fe nanoparticles induce some irreversible reactions and result in poor capacity retention; (3) the SEI film on Fe_3_O_4_ are destroyed due to the large volume change and the repeated formation/decomposition of SEI film^60^. One of the effective methods to overcome these drawbacks is to coat the iron oxides with a carbon shell. Our mesoporous Fe_3_O_4_@CNT possess all of these properties, which facilitate the transmission of lithium ions and electrons, shorten the diffusion time of lithium ions and improve the electron conductivity, and enhance the cycling stability and rate performance. The Fe_3_O_4_@CNT electrode shows excellent electrochemical properties, which can be attributed to the nanoconfinement effect^61^. and the excellent conductivity of CNT as well as the good stability of such special nanostructure. On the other hand, the Fe_3_O_4_@CNT can form a three-dimensional network which not only can accommodate the volume variation on insertion/extraction of lithium ions, but also protect the active materials from severe aggregation^33^. Recently, hybrid composites of iron oxides and CNT as anode materials for LIBs have been reported in various morphologies. Wang *et al*. assembled carbon-coated α-Fe_2_O_3_ hollow nanohorns on the CNT backbone for LIBs^61^. When cycled at high rate of 1–3 C, comparable capacities of 420–500 mAhg^−1^ to those observed here can be maintained. Yu *et al*. reported a hybrid material of CNT-encapsulated Fe_2_O_3_ nanoparticles for LIBs^38^. Electrochemical testing shows that a stable capacity of 335 mAhg^−1^ can be achieved when the current density is 1.2 C. Very recently, Chen and co-workers reported a unique composite with tiny Fe_3_O_4_ dispersed into ~54.6% carbon via a high pressure and temperature based solvothermal route with a capacity of 610 mAh g^−1^ after 100 cycles^62^. Herein, a high capacity of 684.1 mAhg^−1^ can be obtained at 2.0 C on the current mesoporous Fe_3_O_4_@CNT anode. When cycled at high rate 5 C, a capacity of 541.5 mAhg^−1^ can be maintained. Even at 10 C, a similar capacity of 432.2 mAhg^−1^ can still be obtained. Compared with the reported data, the reported mesoporous Fe_3_O_4_@CNT is not the anode that offers the highest discharge capacity, but is comparable with the state-of-the-art of Fe_3_O_4_ based anode. Such mesoporous Fe_3_O_4_@CNT anodes have excellent rate performance ascribed to its interconnected porous nanostructures, which offers a new route for high rate anode.

In order to evaluate the electrochemical dynamical behavior, an electrochemical impedance spectroscopy (EIS) analysis was performed, as shown in Fig. 7e. The EIS spectra of mesoporous Fe_3_O_4_@CNT before and after 50 cycles have a quasi-semicircle in the high frequency region and an inclined linear part at low frequency. The depressed capacitive arc was related to the charge transfer resistance, and the sloped line was attributed to the Warburg impedance that is derived from lithium ion diffusion^27^. It is clearly seen that the diameter of capacitive arc after 50 cycles is smaller than that of the samples before cycles, indicating that the electrical conductivity of mesoporous Fe_3_O_4_@CNT improved after cycling. The small diameter of capacitive arc after cycling indicates the low charge transfer resistance, which will improve the electron kinetics in the electrode materials^18^. Figure 7f shows the HRTEM image of the active materials (Fe_3_O_4_@CNT) after 50 cycles at a rate of 2 C. It is obvious that the Fe_3_O_4_ nanoparticles are still embedded in the CNT. These results indicated the excellent electrochemical properties of the synthesized mesoporous Fe_3_O_4_@CNT. Multi-wall CNT were oxidized into mesoporous structure by a mixed acid solution (HNO_3_ and H_2_SO_4_) at 80 °C. As for CNT, they generally exhibit low dispersibility in water and organic solvents because of their carbonic nature and the presence of van der Waals attraction between the nanotubes due to their hydrophobic properties. After the CNT were oxidized at 80 °C, the CNT are easily dispersed in water and the surface of CNT is porous. The mixed acid solution is strongly oxidising which can cause C-C bond cleavage of local CNT backbone and formation of numerous oxygen-containing groups. The oxidization temperature is very important for the formation of mesoporous CNT. When the temperature was elevated to 120 °C for 20 min, the CNT backbone will be destroyed (Figure S5). Second, when the oxidized CNT were dispersed in the solution containing iron ions (M^z+^, z = 2 and 3, M=Fe), the inside of CNT will be filled completely by the solution based on the capillarity effect, and the oxygen-containing groups on the surface of CNT will act as the active site for the complexation of iron ions by the charge interaction. At this time, the distribution of M^z+^ is in fact composed of two parts: one part of M^z+^ is located in the solution, and another part of M^z+^ is located in inside the CNT. When the NaOH solution was added, the M^z+^ located in the solution will precipitate first. The citrate ions present help control the size of the dispersed M^z+^ precipitates leading to a narrow diameter distribution of particles. When the M^z+^ in solution was gradually consumed, the M^z+^ located at the ends of CNT will be precipitated. Then, the ends of CNT will be blocked with M^z+^ precipitates. However, there will still be some spaces or channels around the blocking M^z+^ precipitates, which facilitate the transmission of the OH^−^ in solution to the M^z+^ in CNT. Driven by the concentration gradient of OH^−^ in solution, the loading level of M^z+^ precipitates located at the ends of CNT will become more and more compact leading to the formation of bigger-size agglomerates. Our experimental results already confirm this fact (see Fig. 2b and Fig. 3a). If the CNT are not oxidized in the mixed acid solution, further filling CNT with iron oxides nanoparticles will be inhibited. Aiming at this challenging problem, we propose this pre-oxidation strategy for the CNT. First, after oxidizing CNT in mixed acid solution, the CNT will become water soluble, which is key factor to fill the CNT with iron oxides nanoparticles *in situ*. In order to avoid the destruction of the CNT backbone, fine control the oxidation parameter is very important. Second, the oxidation of CNT will make the surface of CNT possess numerous defects or pores. These porous structures of CNT will improve the transmission of lithium ions and electrons. Because of the mesoporous structure of CNT, OH^−^ can permeate the carbon shell into the inner of CNT and react with the M^z+^ ions. After that, the inner and outer space of CNT are full of M^z+^ precipitates. When the temperature was elevated to 180 °C for 20 h, M^z+^ precipitates are completely converted into the Fe_3_O_4_ nanoparticles. When the hydrothermal reaction is completed, the cooled solution mixture is composed of Fe_3_O_4_ nanoparticles and mesoporous Fe_3_O_4_@CNT. As for the Fe_3_O_4_ nanoparticles, they are water soluble, small size (Figure S6) and weak magnetic intensity. When a magnet is applied, the Fe_3_O_4_ nanoparticles in solution have no response to the magnet. As for the mesoporous Fe_3_O_4_@CNT, they were full of Fe_3_O_4_ nanoparticles (about 66.5 wt%). This endows the mesoporous Fe_3_O_4_@CNT with a relatively strong magnetic intensity (12.1 emu/g). Therefore, the mesoporous Fe_3_O_4_@CNT are easily isolated from the solution uisng magnet within a few seconds. After washing the mesoporous Fe_3_O_4_@CNT with deionized water five times, the Fe_3_O_4_ nanoparticles in solution, which are not inside the CNT, are completely removed. The efficacy in generating large scale mesoporous Fe_3_O_4_@CNT arises from the capillarity effect, and the mesoporous Fe_3_O_4_@CNT can be obtained on a high scale (about 1.42 g per one-pot, 50 mL hydrothermal reactor). Compared with the conventional method using Al_2_O_3_ membrane as template to fill CNT with iron oxides, our current strategy is very effective, low cost, high filling level and easy large scale production.

## Conclusions

An effective method for *in situ* filling of multi-wall CNT (diameter of 20–40 nm, length of 30–100 μm) with ultrafine Fe_3_O_4_ nanoparticles (8–10 nm) has been demonstrated. The loading level of CNT, in terms of the weight ratio of Fe_3_O_4_ nanoparticles, can reach 66.5 wt% which is significantly higher than the highest reported value (51.8 wt%). The prepared Fe_3_O_4_@CNT exhibited mesoporous properties, and can be obtained on a large scale (~1.42 g per one-pot). The BET surface area of the mesoporous Fe_3_O_4_@CNT was 109.4m^2^g^−1^, which is much higher than that of the commercial Fe_3_O_4_ (~2 m^2^g^−1^). Even when cycled at a high current rate of 20 C, a high capacity of 275.2 mAhg^−1^ could still be achieved. The advantages for the present Fe_3_O_4_@CNT composite compared with that of most previous Fe_3_O_4_/carbon hybrid electrodes are that they facilitate charge transport, maintain the electrode integrity, and endow the electrodes with high capacity, high rate performance and excellent cycling stability showing they are promising anode material for LIBs.

## Additional Information

**How to cite this article**: Gao, G. *et al*. Ultrafine ferroferric oxide nanoparticles embedded into mesoporous carbon nanotubes for lithium ion batteries. *Sci. Rep.* **5**, 17553; doi: 10.1038/srep17553 (2015).

## Supplementary Material

Supplementary Information

## Figures and Tables

**Figure 1:**
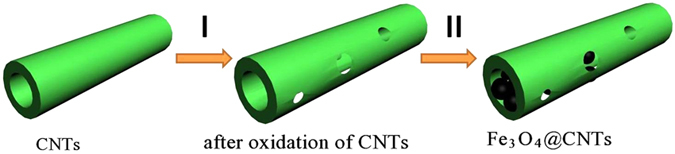
Schematic illustration of the formation process of Fe_3_O_4_@CNT: (I) oxidizing multi-wall CNT into mesoporous structure using mixed acids; (II) *in situ* filling mesoporous CNT with ultra-small Fe_3_O_4_ by one-pot hydrothermal treatment.
