# Supplementary material for: Ultrafine ferroferric oxide nanoparticles embedded into mesoporous carbon nanotubes for lithium ion batteries
Source: Sci Rep. 2015 Dec 3;5:17553. doi: 10.1038/srep17553 (PMC4668383; doi:10.1038/srep17553)
Supplement: Supplementary Information [file srep17553-s1.doc]

**Ultrafine ferroferric oxide nanoparticles embedded into mesoporous carbon nanotubes for high rate lithium ion batteries**

*Guo Gao^a,*^, Qiang Zhang^b^, Xin-Bing Cheng^b^, Joseph G. Shapter^c^, Ting Yin ^a^, Rongjin Sun^a^ and Daxiang Cui^a,*^*

*^a^ Institute of Nano Biomedicine and Engineering, Department of Instrument Science and Technology, Key Laboratory for Thin Film and Microfabrication Technology of Ministry of Education, School of Electronic Information and Electrical Engineering, Shanghai Jiao Tong University, Shanghai, 200240, China.*

*^b^ Beijing Key Laboratory of Green Chemical Reaction Engineering and Technology, Department of Chemical Engineering, Tsinghua University, Beijing, 100084, China.*

*^c^ School of Chemical and Physical Sciences, Flinders University, Bedford Park, Adelaide 5042, Australia.*

Corresponding authors. Fax:+86 21 34206886, E-mail addresses: [guogao@sjtu.edu.cn](mailto:guogao@sjtu.edu.cn) (G. Gao), and [dxcui@sjtu.edu.cn](mailto:dxcui@sjtu.edu.cn) (D. Cui)


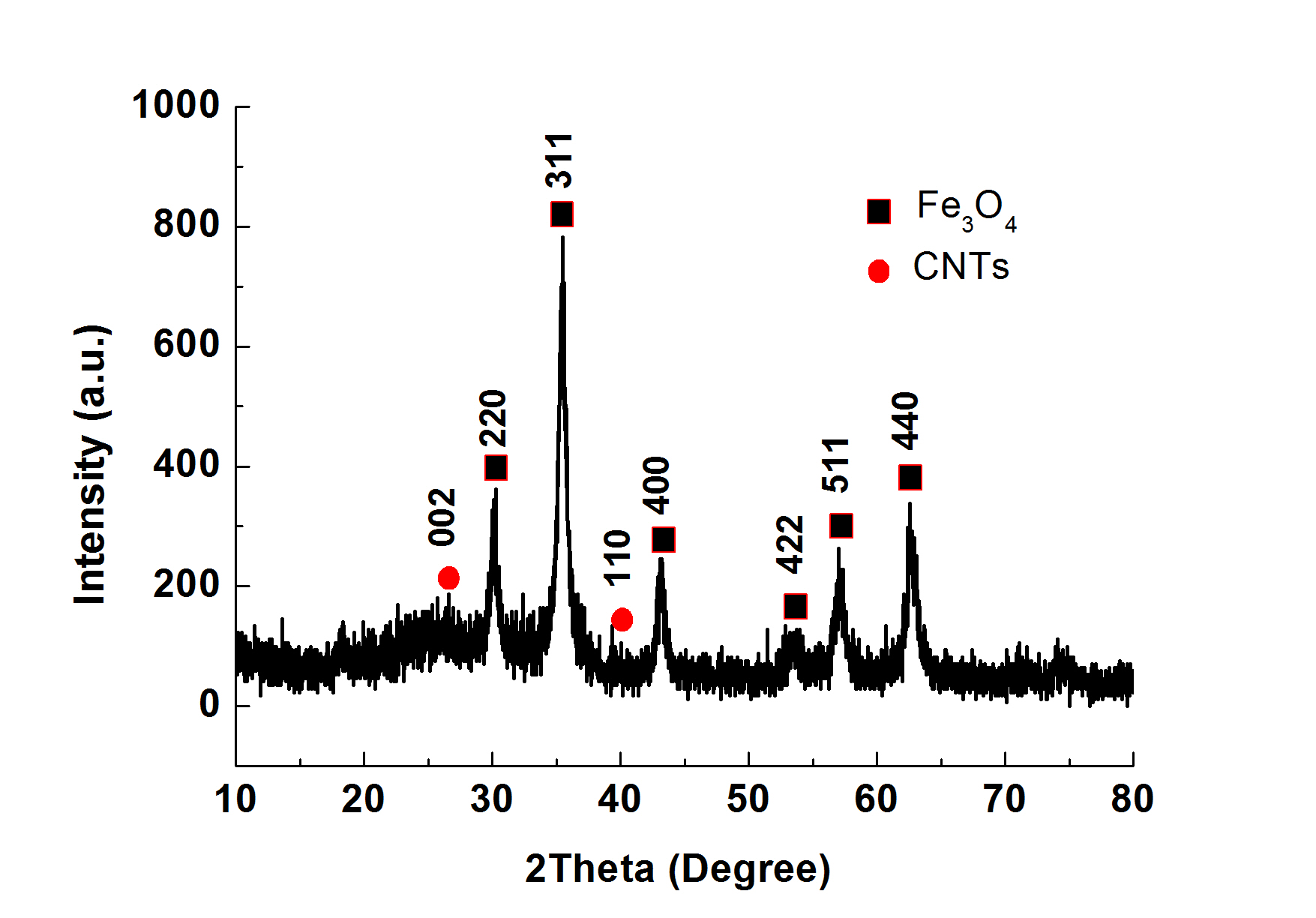


Figure S1 XRD patterns of the synthesized Fe_3_O_4_@CNT.

The average crystalline size was calculated by the Scherrer’s diffraction equation, namely D=Kλ/βcosθ. As for the (311) plane, 2θ=35.48, β=0.79×3.14/180, K=0.89 and λ=0.15405 nm. According to these values, the average crystalline size is ~10.4 nm.


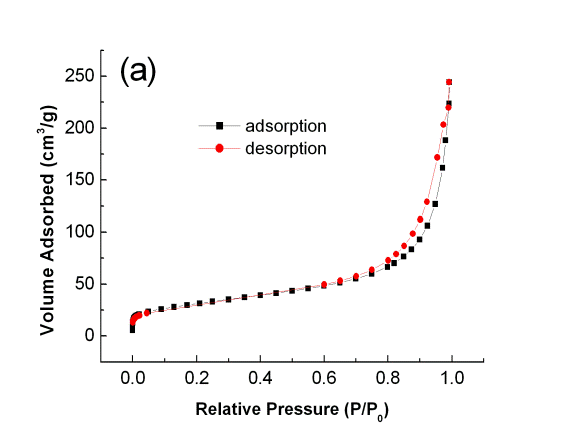

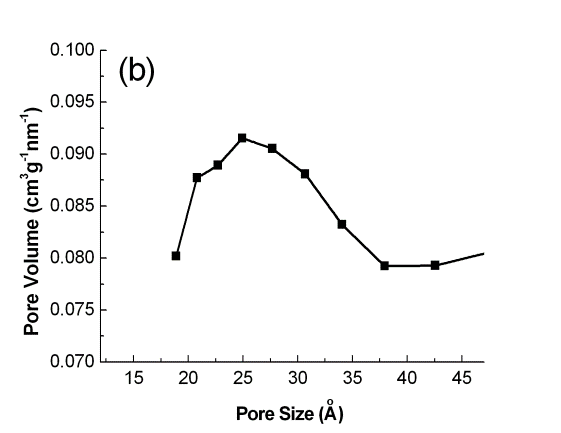


Figure S2 Nitrogen adsorption and desorption isotherms of Fe_3_O_4_@CNT (a), and pore size distribution of Fe_3_O_4_@CNT (b).


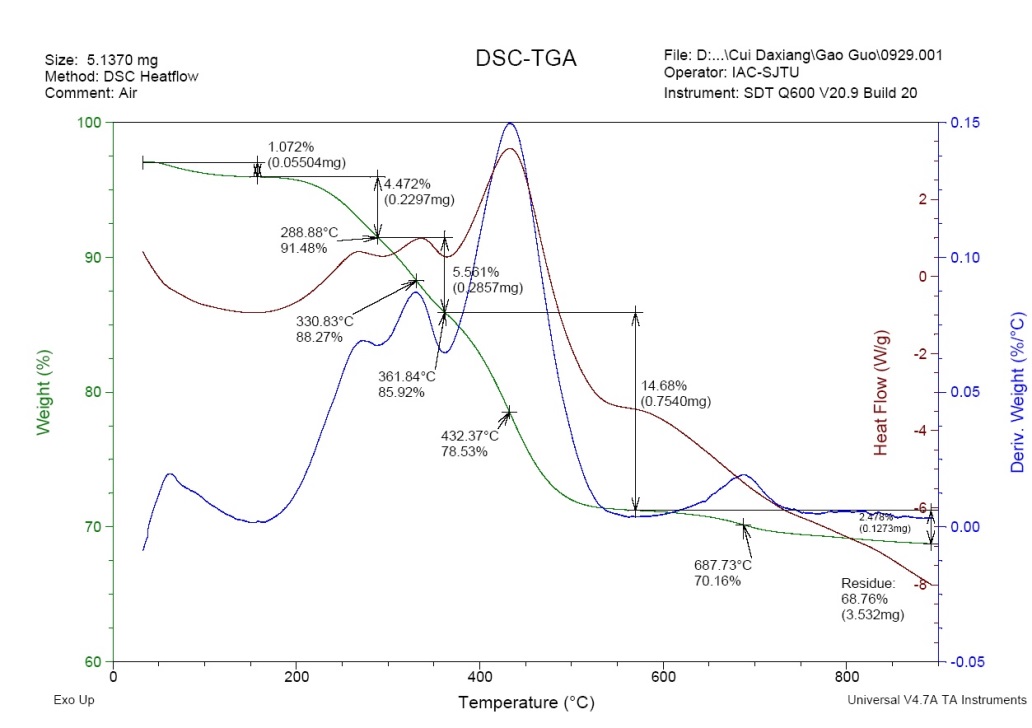


Figure S3 TGA curve of the Fe_3_O_4_@CNT composites measured in air.

In order to evaluate the filling level of CNT by Fe_3_O_4_ nanoparticles, the synthesized Fe_3_O_4_@CNT were measured by TGA test in air, as shown in Figure S3. It is known that CNT cannot be removed in N_2_ gas protection, only oxygen functional groups on the surface of oxidized CNT can be removed. Generally, CNT can be removed over 600 ^o^C in air. On the other hand, the oxidation of Fe_3_O_4_ to Fe_2_O_3_ in air (4Fe_3_O_4_ + O_2_ → 6Fe_2_O_3_) will also lead to the weight increase of samples during the TGA test. Therefore, we must consider both the weight decrease of CNT and the weight increase from the oxidation of Fe_3_O_4_ to Fe_2_O_3_ during the TGA test in air. In order to avoid this complex calculation process, we select the Fe element for our calculation. The initial weight of Fe_3_O_4_@CNT is 5.1370 mg. After TGA test, the Fe_3_O_4_ components will be completely oxidized to Fe_2_O_3_, and CNT will be removed. The residue (Fe_2_O_3_) weight is 3.532 mg (equal to 0.0221 mol). According to the equation of 4Fe_3_O_4_ + O_2_ → 6 Fe_2_O_3_, the initial Fe_3_O_4_ is 0.01473 mol, which is equal to 3.4174 mg. Therefore, the filling level of CNT by Fe_3_O_4_ nanoparticles is about 66.5%.


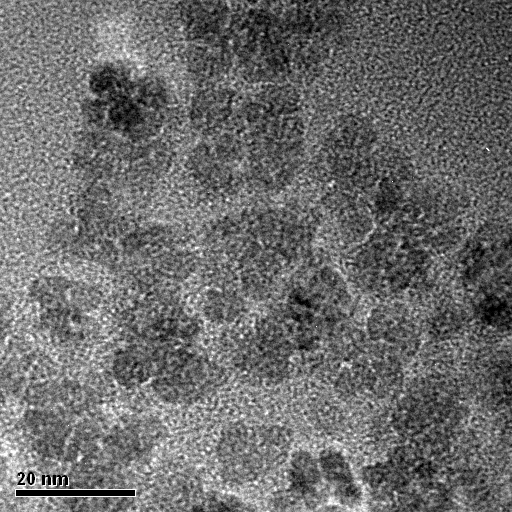


Figure S4 TEM image of the synthesized Fe_3_O_4_ nanoparticles used for LIBs.


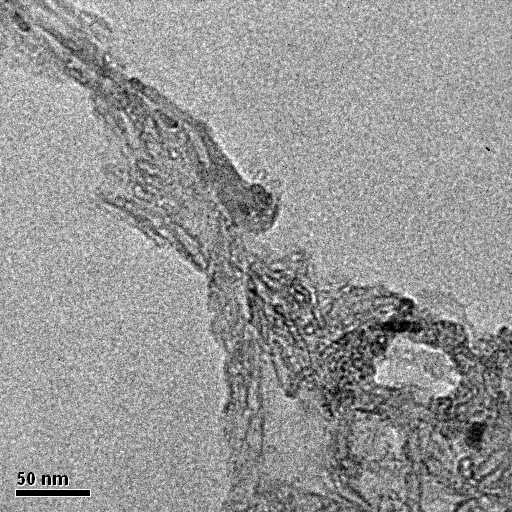

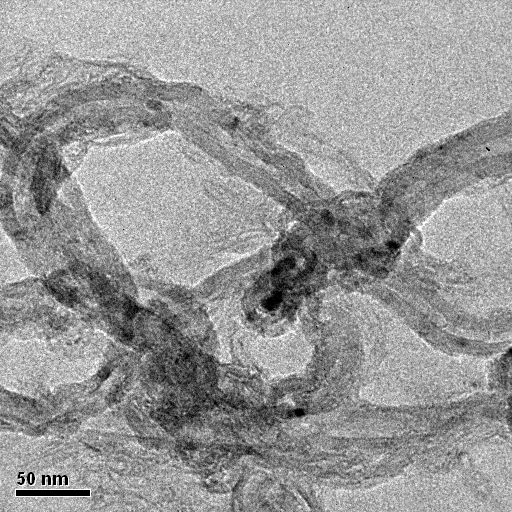


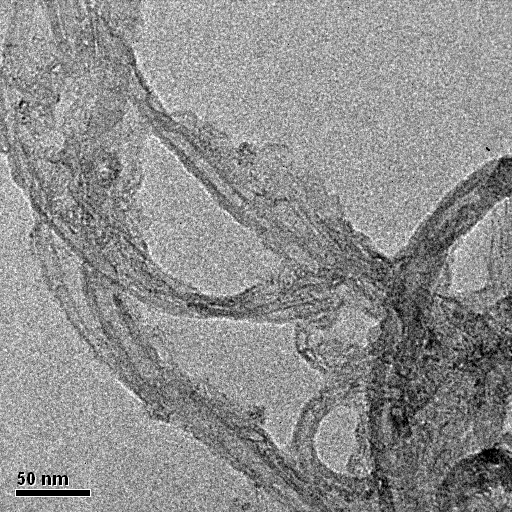

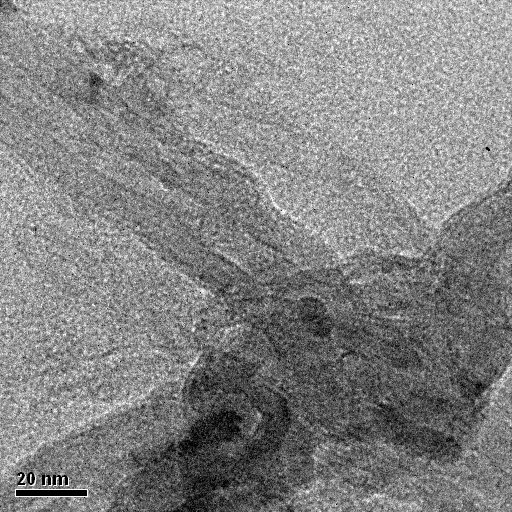


Figure S5 TEM images of the CNT after oxidizing at 120 ^o^C for 20 min.


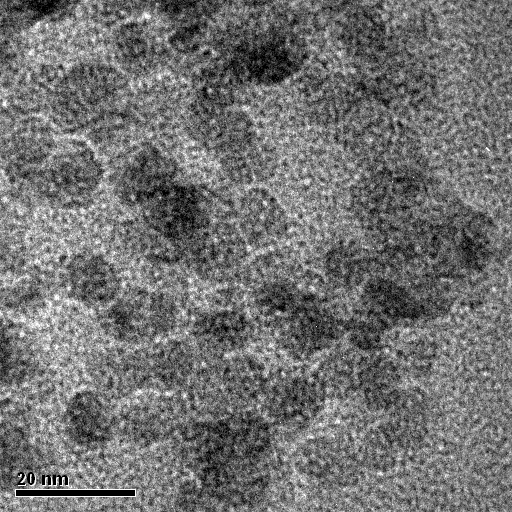


Figure S6 TEM image of the byproduct Fe_3_O_4_ nanoparticles during the synthesis of Fe_3_O_4_@CNT composites.
